# A social network analysis in dynamic evaluate critical industries based on input-output data of China

**DOI:** 10.1371/journal.pone.0266697

**Published:** 2022-04-07

**Authors:** Can Wang, Huipeng Yang

**Affiliations:** School of Business Administration, Zhongnan University of Economics and Law, Wuhan, China; University of Defence in Belgrade, SERBIA

## Abstract

As the Chinese economy grows, the imbalance of industrial structure is prominent, and the optimization of industrial structure has become an urgent problem. Evaluation of industry is an important step in industry optimization. To this end, this study proposes an integrated evaluation method combining social network analysis (SNA) and the multi-criteria decision making (MCDM) method. Specifically, SNA method are used to calculate indicators, the measurement weights are calculated by the Entropy Weight (EW) Method, and the rank of each industry is determined by the TOPSIS method. Critical industries are identified based on China’s input-output data from 2002 to 2017. The results indicate that Manufacturing Industry and the Metal products have a high evaluation, but the Research and Development have a low evaluation value at all times. According to the results, we suggest that the government should optimize the allocation of resources and promote the transfer of resources to balance industrial development.

## Introduction

After more than 40 years of development, China’s economy has grown rapidly, creating a miracle of world economic development. However, the excessively fast development speed has led to uneven industrial development and formed a situation in which a small number of industries play a leading role [1]. By 2020, China’s textile export volume ranks first, industrial output ranks first, and manufacturing ranks first in the world. However, due to the impact of trade disputes between China and the United States, the United States has imposed sanctions on China on computers, communications, semiconductors, and new materials, which had directly led to China’s shortage of chip supply, the decline in mobile phone sales and automobile production. This makes us realize that industrial imbalance will restrict economic development to some extent. Then, how we should find out the shortcomings of the industry and improve the industrial structure has become an important issue. The SNA will be a commonly used method [2]. This motivates us to build China’s industrial network and explore industrial structure issues to reduce economic losses caused by industrial imbalances.

China has completed in several decades what developed countries have done in hundreds of years. Although the total economic volume has risen sharply, there are still some problems in the industrial structure, such as unbalanced industrial development, low utilization of resources between industries, and low industrial aggregation [3, 4]. Due to a large number of industries, large economic aggregates, and complex relationships among industries, it is difficult to judge which industries should be improved or protected based on industrial economic data. When formulating industrial policies, it is difficult to determine which industries need to be supported or optimized to improve the industrial structure. Some traditional qualitative and quantitative methods, like Analytic Hierarchy Process (AHP) and Analytic Network Process(ANP) [5], are either subjective or require large amounts of data. The difficulty in solving these problems is the lack of effective methods to assess the importance of the industry.

There are two challenges in evaluating the criticality of industries. Firstly, the evaluation framework of critical industries should be systematic and all-inclusive, which cannot be directly measured based on one or several indicators. Secondly, with the development of China’s economy, the industry is undergoing constant transformation and upgrading, the structure of the industry is changing dynamically. Therefore, key industries will also change over time. A method for evaluating key industries in a dynamic network is needed. Hence, the purpose of this study is how to evaluate the importance of industry, and what are the structural differences of Chinese industry.

To solve these problems, this study develops a dynamic industrial network model based on China’s input-output data in different periods to promote the evaluation of critical industries. Due to the multi-dimensional nature of network characteristics, we analyze the industrial structure through overall indicators and node indicators. The overall characteristics were measured by network density, network aggregation, and network efficiency. The node characteristics were measured by degree centrality, betweenness centrality, and PageRank. In addition, the influence coefficient and induction coefficient are introduced as supplementary indicators of node measurement. Considering the heterogeneity of nodes, the importance of each indicator is uncertain. Therefore, the EW method is adopted to calculate the weight of the criterion, which can avoid the subjectivity of traditional calculation methods. Given the weight of measurements, the evaluation value of each industry at different time points is obtained through TOPSIS, and the dynamic evolution process of critical industries is analyzed.

This study mainly has the following three contributions. First, an integrated evaluation method is developed for the industry by the EW method and TOPSIS method. The study clearly describes the characteristics of Chinese industry from the whole and part respectively. Moreover, the second contribution is to expand the literature concerning MCDM. Compared with previous literature, we explore the construction of decision models from the social network perspective, which lays a good foundation for future research. Third, the identification of critical industries provides a theoretical basis for policy designation. The Manufacturing and Industry, and the Metal products occupy an important position in the industrial structure. The government should increase investment and support for high-tech industries and service-oriented industries to optimize the industrial structure.

## Literature review

Based on the input-output table, this study assessed critical industries by applying MCDM and SNA. Then, we will chiefly retrospect three aspects of the literature on MCDM, SNA, and applications of social network methods in input-output analysis.

### Multi-criteria decision making

MCDM method is a decision support tool for problems containing multiple and conflicting objectives [6]. It has been used in many fields, such as supply chain management [7], energy [8], waste management [9], urban development [10]. [7] used three MCDM methods to select suppliers, the results showed that all the methods provided highly correlated results, with no significant difference in the resulting position rankings. [11] proposed an environmental vulnerability assessment model based on the EW method to provide a reference for the formulation of environmental protection and related policies in China. [8] establish the objective and measurable patterns to evaluate the shallow geothermal energy implementation through EW method and TOPSIS method. [12] developed TOPSIS and five different strategies to optimize the electro-discharge machining process. [13] illustrate a new hybrid methodology for the selection of offshore wind power station locations based on MCDM.

### Social network analysis

SNA is a quantitative analysis method developed by sociologists based on mathematical methods and graph theory [14]. SNA has been used widely in the social and behavioral sciences, as well as in economics, marketing, and industrial engineering at present. Some scholars have used SNA methods for assessment [15–18]. For instance, [19] used SNA to estimate the quality of the journal. [20] applied SNA to identify schedule risks based on housing production in Hong Kong. [21] examined the risk factors of Infrastructure Projects from the perspective of the social network. In addition, some scholars combine SNA with other research methods. [22] developed an integrated method to evaluate China’s air quality standards by combining SNA, EW method, and TOPSIS method. After that, [23] used SNA and TOPSIS method to dynamic assess critical drafting units of air standards in China. [24] applied SNA and simulation to identify critical risk factors of bridges and tunnels through the accident-related data in China’s bridge-and-tunnel hybrid projects.

### Applications of social network method in input-output analysis

The input-output table can reveal interconnection and balanced proportional relationships among different industries or departments in a certain period [25, 26]. The combination of the SNA method and the input-output table is more and more favored by scholars. [27] made a digraph based on the inter-industry input-output data and studied the industrial association in Washington by using subgraphs and degrees. Some scholars also used different methods to extract the strong correlation between industries to construct the industrial network model [28–30]. [31] employed the SNA to analyze the industrial network. [32] established industrial networks based on the input-output data of more than 20 countries, studied the inter-industry relations, and found that these network structures followed the Weber distribution and had similar community structures. [33] combined input-output analysis and SNA to investigate changes in industrial structure. The results indicated that China’s industrial structure was constantly improving, and the connections between different industries were increasing gradually. [34] estimated the spatial network structure of carbon transfer by multiregional input-output data and SNA method. [35] used SNA to analyze carbon emission transfer network structure by interprovincial input-output tables.

Although the prior literature applied SNA to input-output analysis is extensive, there is no integrated model that can effectively identify critical industries from the social network perspective. The previous limitations motivate us to assess critical industries in the industry network to alleviate the risk of economic restrictions.

## Materials and methods

### A dynamic industrial network model

This study only analysis strong relationships between industries taking into account the diversity of industries and the complexity of relationships. We introduce the Weaver- Thomas (WI) index to regulate the strong and weak threshold value of the relationship [36], to construct the industrial complex network model. The WI index identifies the key elements of a distribution sequence by establishing a series of hypothetical distributions that work with an actual distribution [37]. The calculation steps are as follows:

If there are n industries, *x*(*i*, *j*) represent the consumption coefficient for the products in the industry of *i* in the process of production in industry j. Set *x*(1, *j*), *x*(2, *j*), …, *x*(*n*, *j*) is arranged in order from the largest to the smallest, and the WI index of the *i* term coefficient of the *j* industry is:

$$\begin{array}{c} \begin{array}{c} WI\left(i,j\right)=\sum_{i=1}^{n}{\left[s\left(e,j\right)-100\times \frac{x(e,j)}{\sum_{i=1}^{n}x(i,j)}\right]}^{2}\\ s\left(e,j\right)=\{\begin{array}{cc} 100/i & \left(e\leq i\right)\\ 0 & \left(e≻i\right) \end{array} \end{array} \end{array}$$ (1)

The study assumes that *WI*_*j*_ is the minimum in *WI*(1, *j*), *WI*(2, *j*), *WI*(3, *j*) .…., *WI*(*n, j*). *WI*_*j*_ represents the WI index of the *j* industry. If the consumption coefficient *x*(*i*, *j*) is greater than *WI*_*j*_, there is a strong correlation between the two industries. Based on the WI index method, using the consumption matrix data, the threshold values *WI*_1_,*WI*_2_ and *WI*_3_….,*WI*_*n*_ of each column, are obtained respectively. We use the threshold to construct a 0–1 matrix *m*(*i*, *j*):

$$\begin{array}{c} \left\{ \begin{array}{cc} m(i,j)=1 & \left(x\left(i,j\right)\geq WI_{j}\right)\\ m(i,j)=0 & \left(x\left(i,j\right)≺WI_{j}\right) \end{array}\right. \end{array}$$ (2)

When *m*(*i*, *j*) = 1, there is a link between the two industries. When *m*(*i*, *j*) = 0, there is no link between the two industries. To facilitate the computation of node measurements, we define the matrix *M*^*t*^ = {*N*^*t*^, *S*^*t*^}, where *t* represents the time, *N*^*t*^ is the set of *n*^*t*^, and *S*^*t*^ is the set of *m*^*t*^ directed edges, namely the relationship between industries.

### Evaluation of industry network

(1) Network density: Network density is used to evaluate the extent of the inter-industry correlation in the network [38]. The greater the number of association relationships in the network, the greater the network density [39]. The value of density ranges from 0 to 1, and the closer it is to 1, the greater the network density is. Assume that the number of links in the network is *L*, and the density is expressed by the formula [35]:

$$\begin{array}{c} D_{}^{t}=\frac{L^{t}}{N^{t}\left(N^{t}-1\right)} \end{array}$$ (3)

(2) Network aggregation: The network aggregation implies the degree of industry aggregation in the network. The higher of network aggregation, the more concentrated the network industry is. Network aggregation ranges from 0 to 1, and the closer it is to 1, the more concentrated the industry is. It can be measured by the average clustering coefficient [40]. The Average Clustering Coefficient is the mean value of individual coefficients which can be expressed as follows:

$$\begin{array}{c} CC^{t}=\frac{1}{N}\sum_{i=1}^{N}\frac{{\eta_{i}}^{t}}{{\psi_{i}}^{t}\left({\psi_{i}}^{t}-1\right)} \end{array}$$ (4)

where $\eta_{i}^{t}$ is the amount of actual edges between industry *i* and its $\psi_{i}^{t}$ neighbors.

(3) Network efficiency: Network efficiency represents the speed of network resources and information transmission. The higher the efficiency, the faster the transfer of resources and information. The value of network efficiency is between 0 and 1, and the closer it is to 1, the higher the network efficiency is. The network efficiency can be expressed by the reciprocal of the average path length of the network [41], which is defined by:

$$\begin{array}{c} E^{t}=\frac{1}{\frac{1}{N(N-1)}\sum_{i\neq j}^{N}e_{i,j}^{t}} \end{array}$$ (5)

where $e_{i,j}^{t}$ represents the shortest path length between industry *i* and *j*.

### Evaluation of industry node

(1) Degree centrality: The degree centrality of the industrial network can be measured by in-degree and out-degree [42]. The out-degree represents the count of industries invested and the in-degree represents the count of other industries invested in the current industry. The degree centrality is measured by the sum of in-degree and out-degree. The higher degree of centrality indicates that the industry and other industrial resources exchange more frequently. Based on the definition of the industry matrix *M*^*t*^, the out-degree of industry *i* at time *t* is represented by $dc\_out_{i}^{t}$, is further defined by:

$$\begin{array}{c} dc\_out_{i}^{t}=\sum_{j=1}^{N}m_{ij}^{t} \end{array}$$ (6)

Similarly, $dc\_in_{i}^{t}$ in represents the in-degree of industry *i*, which is defined as:

$$\begin{array}{c} dc\_in_{i}^{t}=\sum_{j=1}^{N}m_{ji}^{t} \end{array}$$ (7)

Hence, the degree centrality of industry *i* is defined as:

$$\begin{array}{c} DC_{i}^{t}=dc\_out_{i}^{t}+dc\_in_{i}^{t} \end{array}$$ (8)

(2) Betweenness centrality: The betweenness centrality denotes the ratio of the number of paths that pass the node to the total number of all the shortest paths in the network [43–45]. In the industry network, the industry with high betweenness centrality has a vigoroso capability to hold assets or intelligence spread. The betweenness centrality can be expressed as follows:

$$\begin{array}{c} BC_{i}^{t}=\frac{\sum_{s\neq u\neq i}^{N}\delta_{s,u}^{i}}{v^{t}} \end{array}$$ (9)

Where $\delta_{s,u}^{i}=1$ if a path origin from industry *s* and the finale at *u* at time *t* passes through node *i*; otherwise, $\delta_{s,u}^{i}=0$, and *v*^*t*^ is the amount of the shortest paths in the entire network.

(3) PageRank: PageRank represents the importance of the industry’s position in the network [46]. The PageRank is between 0 and 1, and the industry with high PageRank has an important position in the network. We assumed that industry *j* points to industry *i*, and industry *j* is in the central position and has a lot of resources, then industry *i* will also be affected. Meanwhile, the industry *j* distributes resources evenly to its connected industries, then PageRank is defined as:

$$\begin{array}{c} PR_{i}^{t}=\frac{1-\alpha }{N}+\alpha \sum_{j=1}^{N}\frac{PR_{j}^{t}}{dc\_out_{j}^{t}} \end{array}$$ (10)

Where $PR_{j}^{t}=0$ if industry *j* does not point to the industry *i* at time *t*; otherwise, $PR_{j}^{t}\neq 0$, and *α* is the damping factor, which is generally 0.85.

(4) Influence coefficient: The influence coefficient represents the influence degree of the demand change of industry *i* on other industries [47]. The industry with a high influence coefficient has a stronger pulling effect on the national economy. The influence coefficient is defined as:

$$\begin{array}{c} T_{i}^{t}=\frac{\sum_{i=1}^{N}b_{ij}}{\frac{1}{N}\sum_{i=1}^{N}\sum_{j=1}^{N}b_{ij}} \end{array}$$ (11)

$$\begin{array}{c} \begin{array}{cc} a_{ij}=\frac{x_{ij}}{x_{j}} & \left(i,j=1,2,3\ldots ‥N\right) \end{array} \end{array}$$ (12)

Where *b*_*ij*_ refer to the complete consumption coefficient of industry *j* to industry *i*, *a*_*ij*_ is the direct consumption coefficient of industry *j* to industry *i*, *x*_*ij*_ represents the amount of input from industry *i* to industry *j*, *x*_*j*_ represents the total amount of other inputs to industry *j*. The complete consumption matrix is represented by **B**, and the direct consumption matrix is represented by **A**. The complete consumption matrix ***B*** = (***I*** − ***A***)^−**1**^ − ***I***, which **I** the represents identity matrix. **B** is represented by the matrix as:

$$\begin{array}{c} B=[\begin{array}{cccc} b_{11} & b_{12} & \ldots & b_{1n}\\ b_{21} & b_{22} & \ldots & b_{2n}\\ \ldots & \ldots & \ldots & \ldots \\ b_{n1} & b_{n2} & \ldots & b_{nn} \end{array}]={\left(\left[\begin{array}{cccc} 1 & 0 & \ldots & 0\\ 0 & 1 & \ldots & 0\\ \ldots & \ldots & \ldots & \ldots \\ 0 & 0 & \ldots & 1 \end{array}]-[\begin{array}{cccc} a_{11} & a_{12} & \ldots & a_{1n}\\ a_{21} & a_{22} & \ldots & a_{2n}\\ \ldots & \ldots & \ldots & \ldots \\ a_{n1} & a_{n2} & \ldots & a_{nn} \end{array}\right]\right)}^{-1}-[\begin{array}{cccc} 1 & 0 & \ldots & 0\\ 0 & 1 & \ldots & 0\\ \ldots & \ldots & \ldots & \ldots \\ 0 & 0 & \ldots & 1 \end{array}] \end{array}$$ (13)

(5) Induction coefficient: The induction coefficient indicates the degree to which industry *j* responds to changes in demand of other industries [47]. The industry with a high induction coefficient has a durable role in promoting the national economy and can provide more resources for economic development. The induction coefficient is defined as:

$$\begin{array}{c} Q_{i}^{t}=\frac{\sum_{j=1}^{N}d_{ij}}{\frac{1}{N}\sum_{i=1}^{N}\sum_{j=1}^{N}d_{ij}} \end{array}$$ (14)

$$\begin{array}{c} \begin{array}{cc} c_{ij}=\frac{x_{ij}}{x_{i}+y_{i}} & \left(i,j=1,2,3\ldots ..N\right) \end{array} \end{array}$$ (15)

Where *d*_*ij*_ indicates complete distribution coefficient of industry *i* to industry *j*, *c*_*ij*_ indicates the direct distribution coefficient of industry *i* to industry *j*, *x*_*ij*_ represents the amount of input from industry *i* to industry *j*, *x*_*i*_ represents the total amount of inputs industry *i*, *y*_*i*_ represents the total amount of industry *i* imports. The complete distribution matrix is denoted by **D**, and the direct distribution matrix is denoted by **C**. The complete consumption matrix ***D*** = (***I*** − ***C***)^−**1**^ − ***I***, which **I** represents the identity matrix. **D** is represented by the matrix as:

$$\begin{array}{c} D=[\begin{array}{cccc} d_{11} & d_{12} & \ldots & d_{1n}\\ d_{21} & d_{22} & \ldots & d_{2n}\\ \ldots & \ldots & \ldots & \ldots \\ d_{n1} & d_{n2} & \ldots & d_{nn} \end{array}]={\left(\left[\begin{array}{cccc} 1 & 0 & \ldots & 0\\ 0 & 1 & \ldots & 0\\ \ldots & \ldots & \ldots & \ldots \\ 0 & 0 & \ldots & 1 \end{array}]-[\begin{array}{cccc} c_{11} & c_{12} & \ldots & c_{1n}\\ c_{21} & c_{22} & \ldots & c_{2n}\\ \ldots & \ldots & \ldots & \ldots \\ c_{n1} & c_{n2} & \ldots & c_{nn} \end{array}\right]\right)}^{-1}-[\begin{array}{cccc} 1 & 0 & \ldots & 0\\ 0 & 1 & \ldots & 0\\ \ldots & \ldots & \ldots & \ldots \\ 0 & 0 & \ldots & 1 \end{array}] \end{array}$$ (16)

### Method for industrial evaluation

To evaluate industry importance comprehensively and systematically, a holistic evaluation model is proposed. To be more specific, we build an integrated method by the EW method and TOPSIS method. The weights for several criteria are calculated based on the EW method. These weights are inputted to the TOPSIS method, the rank of each industry is determined according to its results.

As you know, weights can be determined using different methods for instance AHP, Full Consistency Method (FUCOM), Level Based Weight Assessment (LBWA), Criteria Importance Through Intercriteria Correlation (CRITIC), EW. AHP, FUCOM, and LBWA are subjective methods, which are influenced by expert knowledge and experience [48]. CRITIC is an objective approach, but it can change the normalization process of the initial matrix elements and the function for aggregating data that represents values of weight coefficients [49]. Therefore, we choose the EW method to calculate the weight. Firstly, the EW method can overcome subjective measurement errors which some traditional methods such as AHP and ANP cannot do [50, 51]. In addition, the EW method can make full use of raw data information and capture the implicit interaction between each factor, to determine the weight of each factor [8]. Thirdly, the EW method has been successfully applied in many studies, such as environmental assessment [11], water quality assessment [50], energy [8], and road safety management [52].

**Fig 1:**
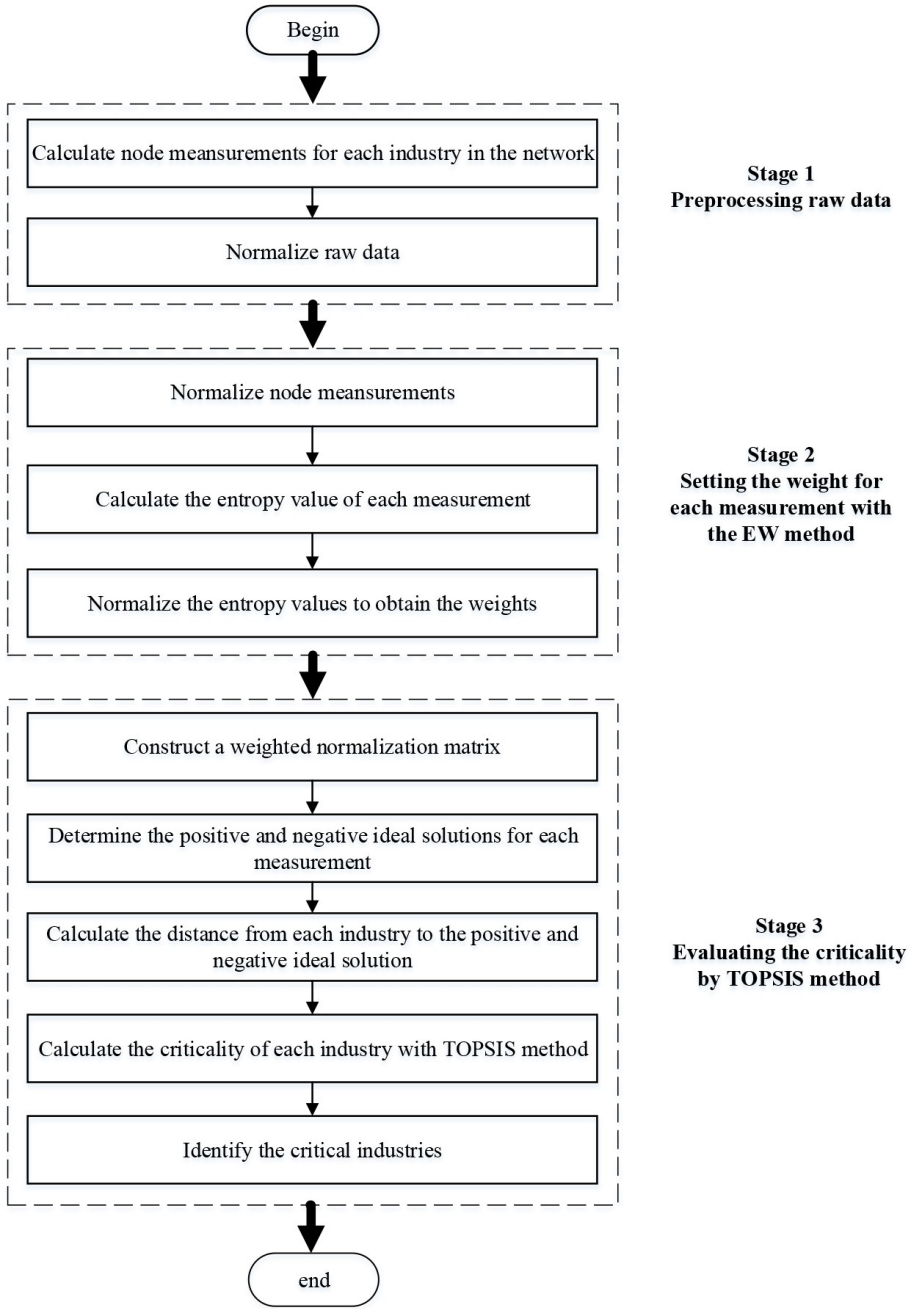
The procedure of the integrated method for industrial evaluation.

Compared with other MCDM methods, such as Decision-making Trial and Evaluation Laboratory(DEMATEL), VIKOR, Multi-Attribute Border Approximation Area Comparison(MABAC), the TOPSIS method has a better result in index ranking [53]. Moreover, TOPSIS is a widely used MCDM method, which has been used by many scholars [8, 12]. The calculation process of TOPSIS is clear and can measure the relative performance of each solution in simple mathematical form [54]. The EW method can effectively improve the performance of TOPSIS through discrete probability distribution [12]. The evaluation accuracy can be enhanced by combining the EW method and TOPSIS method. The overall procedure is shown in Fig 1.

#### Calculation of weight

The EW method can give different weights to different node measurements by objective methods [22]. Based on this weight, we can measure the importance of the industry. The calculation process is illustrated as follows:

**Step 1. Index standardization**. Due to the units of measurement being different, all the measured values are normalized. First, we set up the decision matrix *G*(*γ*_*ij*_) where *γ*_*ij*_ represents the *j*th measurement value of the industry *i*. The standardization of these measurements is described in Formula 17, where $\gamma_{j}^{\text{min}}={\text{min}}_{i=1}^{N}\{\gamma_{ij}\}$ and $\gamma_{j}^{\text{max}}={\text{max}}_{i=1}^{N}\{\gamma_{ij}\}$. Then, we get the standardized matrix *H*(*ε*_*ij*_), where *ε*_*ij*_ represents the *j*th standardized measurement value of the industry *i*.
  $$\begin{array}{c} \varepsilon_{ij}=\frac{\gamma_{ij}-\gamma_{j}^{\text{min}}}{\gamma_{j}^{\text{max}}-\gamma_{j}^{\text{min}}} \end{array}$$ (17)
**Step 2. Entropy estimation**. Evaluate the entropy for the *j*th indicator, which is described in Formulas 18 and 19, where $\lim_{p_{ij}\to 0}p_{ij}\text{ln}p_{ij}=0$ if *p*_*ij*_ = 0.
  $$\begin{array}{c} k_{j}=-\frac{1}{lnN}\sum_{i=1}^{N}p_{ij}\;\text{ln}\;p_{ij} \end{array}$$ (18)
  $$\begin{array}{c} p_{ij}=\frac{\varepsilon_{ij}}{\sum_{i=1}^{N}\varepsilon_{ij}} \end{array}$$ (19)
**Step 3. Weights’ generation**. Assess the weight for the *j*th indicator, which is indicated as:
  $$\begin{array}{c} w_{j}=\frac{1-k_{j}}{N-\sum_{j=1}^{\tau }k_{j}} \end{array}$$ (20)

### Identification of critical industries

TOPSIS is calculated based on the distance to the positive and negative ideal solutions. The specific calculation process is as follows:

**Step 1. Normalized decision matrix**. The normalized decision matrix *ε*_*ij*_ is constructed in Formula 17.
**Step 2. Construction of weighted normalization matrix**. The weighted normalization matrix *V*_*ij*_ is described in Formula 21.
  $$\begin{array}{c} v_{ij}=\varepsilon_{ij}*w_{j} \end{array}$$ (21)
**Step 3. Determination of the positive and negative ideal reference points**. The positive and negative ideal reference points can be outlined as follows:
  $$\begin{array}{c} V^{+}=\left\{\text{max}\left(v_{ij}\right)\right\}\left(j=1,2,\ldots ..,m\right) \end{array}$$ (22)
  $$\begin{array}{c} V^{-}=\left\{\text{min}\left(v_{ij}\right)\right\}\left(j=1,2,\ldots ..,m\right) \end{array}$$ (23)
**Step 4. Calculation of the distances to the positive and negative ideal reference points**. The distance to the positive ideal points is $S_{i}^{+}$, the distance to the negative ideal points is $S_{i}^{-}$.
  $$\begin{array}{c} S_{i}^{+}=\sqrt{\sum_{i=1}^{n}\left(V_{ij}-V_{j}^{+}\right)}\left(i=1,2\ldots .,n\right) \end{array}$$ (24)
  $$\begin{array}{c} S_{i}^{-}=\sqrt{\sum_{i=1}^{n}\left(V_{ij}-V_{j}^{-}\right)}\left(i=1,2\ldots .,n\right) \end{array}$$ (25)
**Step 5. Calculation of the comprehensive ranking index**. The comprehensive ranking index can be calculated as follows:
  $$\begin{array}{c} Z_{i}=\frac{S_{i}^{-}}{S_{i}^{-}+S_{i}^{+}} \end{array}$$ (26)

The higher the value of *Z*_*i*_, the more important industry *i* is.

### Data sources

**Fig 2:**
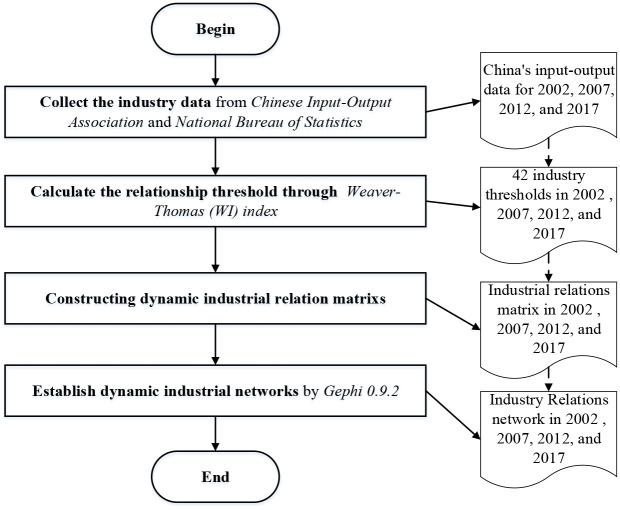
The procedure of data collection and processing.

Data were obtained from the *Chinese Input-Output Association* and *the National Bureau of Statistics*. In these platforms, we can obtain input-output data from national and regions across industries. Since the input-output tables have been published every five years since 1982, they have recently been published successively in 2017. Before 2002, the industry division in the national input-output table was inconsistent, so this study selected the national input-output data of 2002, 2007, 2012, and 2017. These input-output tables divide the industry into 42 and detail information about the industry as shown in S1 Appendix. The entire procedure of data collection and processing is depicted in Fig 2.

## Results

**Fig 3:**
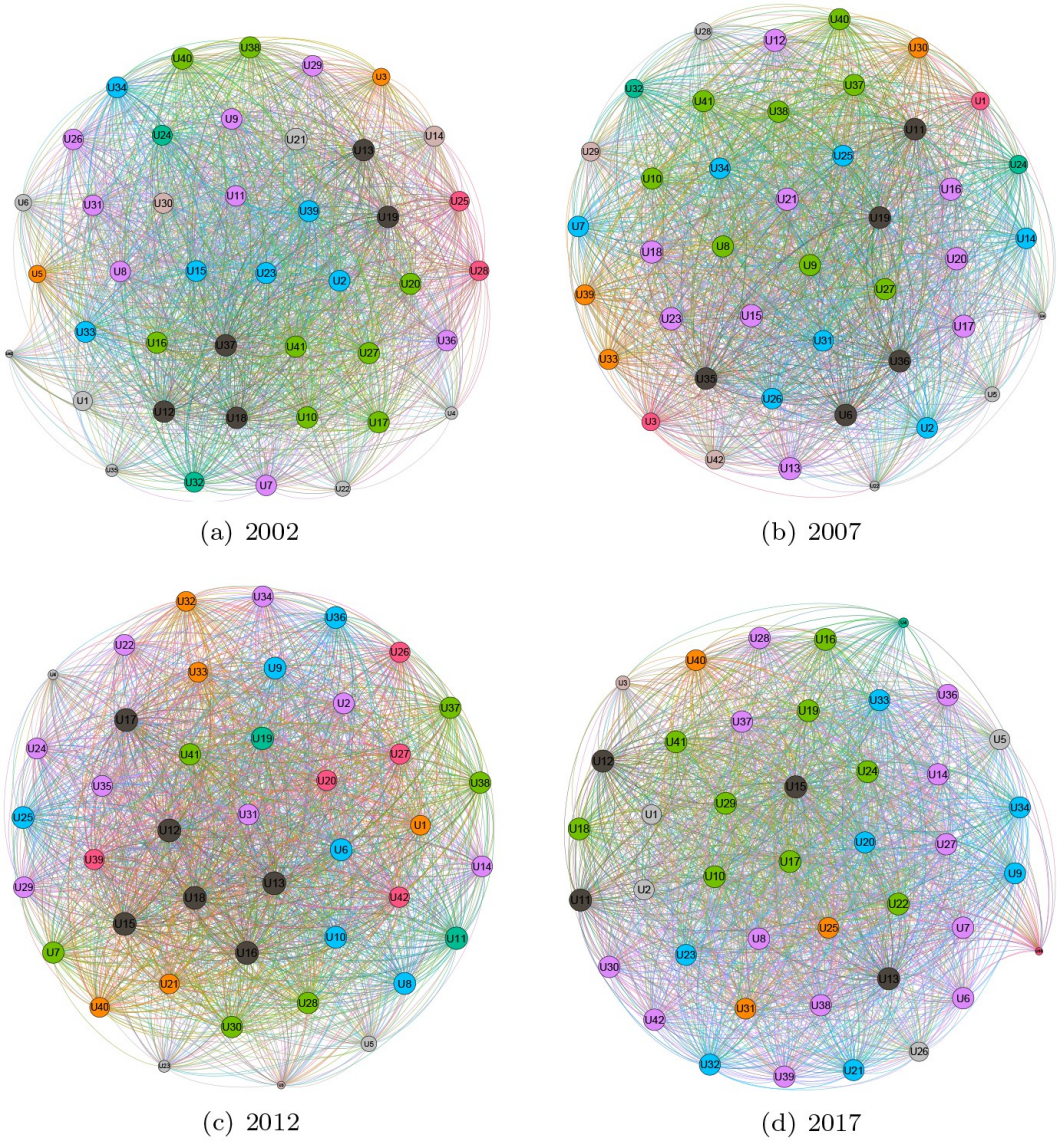
The evolution of the industry network.

**Fig 4:**
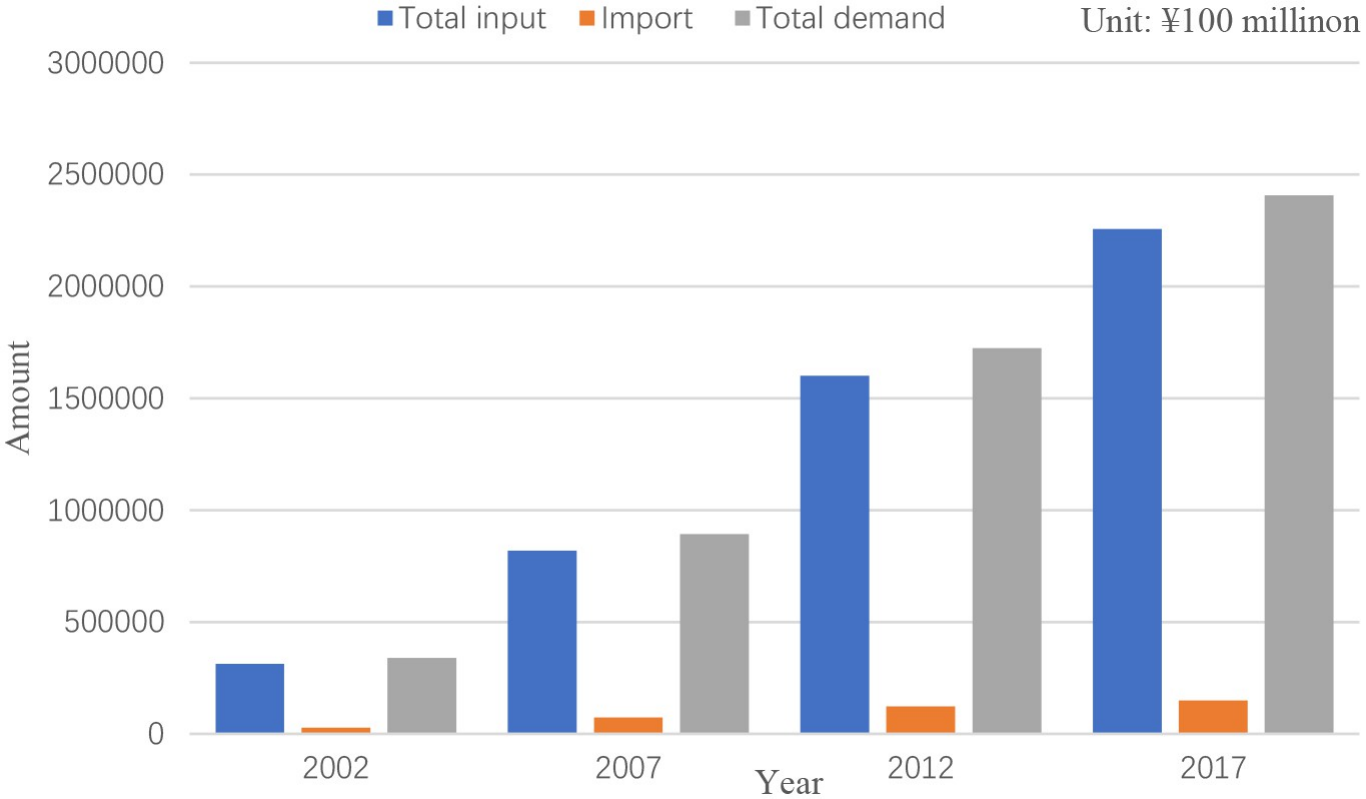
Quantity of total input, import, and total demand annual.

The evolution of the industry network is shown in Fig 3 by Gephi 0.9.2, in which the nodes denote industry, the node labels denote the industry number and the lines denote the interrelationships between industries. Fig 3 shows that there is an obvious trend that the industry network is becoming increasingly complicated each year. From Fig 4, we can also see that the total input, import, and total demand present a rising trend. To further investigate the evolution of the industry network, we will focus on the computational results of the measurements introduced in the previous section. The basic information of industries mentioned in the study is shown in Table 8 in S1 Appendix.

### Overall evaluation results of industry network

As we can see from Table 1 that the network density and network aggregation reached the maximum in 2007, which were 0.952 and 0.953, respectively. The main reason is that industries have gradually developed under the promotion of foreign investment, and the industrial chain has gradually matured, which has increased the exchanges and resource exchange among industries, since China joined the WTO in December 2001. However, the network density and network agglomeration decreased slightly in 2012 and 2017. The main reason is the outbreak of the subprime crisis in the US in 2008 [55]. Affected by the financial crisis, the global economy began to decline, and China was inevitably affected. From Table 1, we can see that network efficiency has been on the rise. The main reason lies in the development of transportation and technology, which has accelerated the speed of resource exchange between industries and improved the ability of industrial information communication.

**Table 1:** Evaluation results of industry network.

Year	Network density	Network aggregation	Network efficiency
2002	0.907	0.911	0.905
2007	0.952	0.953	0.954
2012	0.94	0.94	0.943
2017	0.933	0.933	0.958

### Evaluation results of industry node

#### Degree centrality

The results of the evolution of the top five industries are shown in Table 2. We can see that the top 5 industries have a high degree of centrality which is greater than 80. This indicates that these industries have a high degree of output and input, and there is resource exchange between them and other industries. Moreover, the Chemical products(U12), Non-metallic mineral products(U13) had a great degree of centrality at all times. The manufacturing industry has always had a high degree. Since 2007, the manufacturing industry began to develop into a subdivision industry, gradually becoming Ordinary machinery(U15) and Special equipment(U16). Finally, in response to the national strategy of strengthening cultural power in 2017, the degree centrality of the Printing industry, cultural, educational, and sports goods(U10) in 2017 entered the top five for the first time.

**Table 2:** Top 5 industries with degree centrality.

Rank	2002		2007		2012		2017	
	*u*_*i*_	$DC_{i}^{t}$	*u*_*i*_	$DC_{i}^{t}$	*u*_*i*_	$DC_{i}^{t}$	*u*_*i*_	$DC_{i}^{t}$
1	U21	81	U12	82	U12	82	U11	81
2	U12	80	U13	82	U13	82	U12	81
3	U13	80	U15	82	U15	82	U13	81
4	U18	80	U16	82	U16	82	U15	81
5	U19	80	U17	82	U17	82	U10	80

#### Betweenness centrality

It can be seen that the top 5 industries have a low betweenness centrality which is less than 0.01 except the Scrap waste(U22), which has a degree of 0.0513 from Table 3. We believe that the main reason is that the science and technology of China were relatively backward, and waste treatment and reuse required huge costs and resources in 2002, which in the primary stage of reform and opening up. In addition, the Chemical products(U12), Non-metallic mineral products(U13), and Metal products(U15) had a great degree of centrality in 2007, 2012, and 2017. This indicates that chemical and manufacturing occupy most of the country’s resources. We found that Coke, refined petroleum products, and nuclear fuel (U11) have highest betweenness centrality in 2017. Due to the large consumption of resources and the environment by the development of industrialization, China is now beginning to save resources, the development of new energy as the focus.

**Table 3:** Top 5 industries with betweenness centrality.

Rank	2002		2007		2012		2017	
	*u*_*i*_	$BC_{i}^{t}$	*u*_*i*_	$BC_{i}^{t}$	*u*_*i*_	$BC_{i}^{t}$	*u*_*i*_	$BC_{i}^{t}$
1	U22	0.0513	U12	0.0049	U12	0.0077	U11	0.0091
2	U21	0.0091	U13	0.0049	U13	0.0077	U12	0.0091
3	U18	0.0084	U15	0.0049	U15	0.0077	U13	0.0091
4	U19	0.0084	U16	0.0049	U16	0.0077	U15	0.0091
5	U20	0.0082	U17	0.0049	U17	0.0077	U14	0.0085

#### PageRank

Table 4 indicates the result of PageRank. It can be seen that the PageRank of all industries is low, which indicates the distinction of industrial status is not obvious. Meanwhile, it can be found that in 2007, 2012, and 2017, these industries that had high PageRank include the Chemicals products(U12), Non-metallic mineral products(U13), and Metal products(U15). However, the R&D and other business activities(U35) had the highest PageRank in 2017. This is mainly because China put forward the strategy of shifting from “Made in China” to “Created in China” in 2015. Therefore, more and more resources are invested in research and development and experiment, and it shows an increasingly critical role in the industry.

**Table 4:** Top 5 industries with PageRank.

Rank	2002		2007		2012		2017	
	*u*_*i*_	$PR_{i}^{t}$	*u*_*i*_	$PR_{i}^{t}$	*u*_*i*_	$PR_{i}^{t}$	*u*_*i*_	$PR_{i}^{t}$
1	U22	0.04	U12	0.02	U12	0.03	U35	0.03
2	U21	0.03	U13	0.02	U13	0.03	U11	0.03
3	U18	0.02	U15	0.02	U15	0.03	U12	0.03
4	U19	0.02	U16	0.02	U16	0.03	U13	0.03
5	U12	0.02	U17	0.02	U17	0.03	U15	0.03

#### Influence coefficient

The influence coefficient of the top five industries at four stages is displayed in Table 5. We see that the influence coefficient of all the industries was about 1.5. Transport equipment(U18), Electric equipment and machinery(U19), and Electronic and telecommunications equipment(U20) had a great influence coefficient at all times. This indicates that manufacturing has always been the foundation of China’s industrial development, which needs a lot of resources. Furthermore, we also observed that the Garments and other fiber products(U8) has a great influence coefficient of 1.47 in 2017. This is mainly because China’s textile exports have ranked first in the world since 2016. Hence, Garments and other fiber products(U8) need more resources to meet the growing demand.

**Table 5:** Top 5 industries with influence coefficient.

Rank	2002		2007		2012		2017	
	*u*_*i*_	$T_{i}^{t}$	*u*_*i*_	$T_{i}^{t}$	*u*_*i*_	$T_{i}^{t}$	*u*_*i*_	$T_{i}^{t}$
1	U19	1.81	U19	1.64	U20	1.56	U20	1.71
2	U20	1.61	U18	1.51	U19	1.49	U19	1.47
3	U18	1.57	U20	1.5	U18	1.43	U8	1.43
4	U17	1.56	U17	1.5	U16	1.41	U21	1.42
5	U15	1.54	U15	1.39	U24	1.4	U18	1.41

#### Induction coefficient

The results on the induction coefficient are presented in Table 6. It can be seen that the induction coefficient of all industries at four stages is about 2.0. Industries with high sensitivity at each time point did not change much. Coal mining and dressing (U2), Petroleum and natural gas extraction(U3), and Metals mining and dressing (U4) had a great induction coefficient at all times [56]. These industries are resource exploitation industries, providing other industries with necessary energy. Therefore, they will change with the needs of other industries.

**Table 6:** Top 5 industries with induction coefficient.

Rank	2002		2007		2012		2017	
	*u*_*i*_	$Q_{i}^{t}$	*u*_*i*_	$Q_{i}^{t}$	*u*_*i*_	$Q_{i}^{t}$	*u*_*i*_	$Q_{i}^{t}$
1	U4	2.33	U3	2.26	U2	2.23	U2	2.21
2	U3	2.31	U2	2.17	U3	2.18	U3	2.16
3	U22	2.15	U4	2.05	U4	2.07	U4	2.12
4	U11	1.78	U23	2.01	U23	1.96	U24	1.82
5	U2	1.73	U22	1.87	U25	1.88	U22	1.72

### Result of industrial evaluation

Calculated by the integrated method, all industries can be assessed. The results are shown in Table 7. It can be seen from Table 7 that the results calculated by the EW method are similar to those obtained by the EW-TOPSIS. However, compared with the integrated evaluation method, the result of the EW method is more susceptible to the influence of a single index, and the unstandardized data will have a great impact on the weight. Therefore, the result of the EW-TOPSIS calculation will be more stable. We can see that the Manufacturing and Industry have high evaluation value at all times, for instance, the Metal products(U15), Chemicals products(U12), and Electric equipment and machinery(U18). It shows that Industry and Manufacturing are still important pillar industries in China, and a lot of resources have been invested in them. In addition, the industries with low evaluation values are Education(U39), Wholesale and retail(U28), and R&D and other business activities(U35). It indicates that the service sector and technology are still weak spots in China’s economic development. Although the maximum gap between the assessed values of industries is narrowing, the gap is still large, remaining at about 0.7.

**Table 7:** Top 5 critical industries.

Rank	2002				2007				2012				2017			
	EW		EW-TOPSIS		EW		EW-TOPSIS		EW		EW-TOPSIS		EW		EW-TOPSIS	
	*u*_*i*_	*Z*_*i*_	*u*_*i*_	*Z*_*i*_	*u*_*i*_	*Z*_*i*_	*u*_*i*_	*Z*_*i*_	*u*_*i*_	*Z*_*i*_	*u*_*i*_	Z	*u*_*i*_	*Z*_*i*_	*u*_*i*_	*Z*_*i*_
1	U22	21.13	U22	0.81	U23	6.51	U23	0.90	U12	6.31	U12	0.88	U12	12.38	U12	0.89
2	U21	6.61	U12	0.29	U12	6.42	U12	0.85	U15	6.24	U15	0.84	U11	12.37	U11	0.87
3	U19	6.39	U19	0.28	U15	6.35	U15	0.81	U16	6.21	U11	0.83	U15	12.34	U14	0.87
4	U18	6.38	U4	0.28	U18	6.31	U14	0.79	U13	6.20	U13	0.82	U13	12.30	U15	0.86
5	U20	6.23	U14	0.28	U20	6.31	U13	0.78	U18	6.16	U16	0.81	U14	11.90	U13	0.84

## Discussion

Through the analysis of the overall structure of the industry and the node measurement, the critical industries are identified by using the EW method and TOPSIS method, and the following views can be obtained:

1. China’s total input and total demand reveal an upward trend. However, the network density and network aggregation of the industry decreased slightly, which indicates that the connectivity of the industry relationship is still insufficient. There are two main reasons for this. Firstly, due to rapid economic development and insufficient planning, a reasonable industrial cluster cannot be formed. Secondly, affected by the world economic crisis and trade disputes between China and the United States, the exchange of resources and information transmission between Chinese industries has weakened in recent years. In addition, industrial efficiency is constantly improving, since the popularity of connectivity and technological progress.
2. The top five industries at four points have hardly changed. The indicators of manufacturing and industry, namely the degree centrality, betweenness centrality, and PageRank were high at four stages. Resource-intensive industries, such as Transport equipment(U18) and Electronic and telecommunications equipment(U20) had a great influence coefficient at all times. The Energy production industry, for example, the Coal mining and dressing (U2) and Metals mining and dressing (U4) had a great induction coefficient at all times.
3. Resource consumption is the main driving force of economic development. We observed that the Manufacturing and Industry, and the Metal products have high evaluation value at all times. This shows that China is still a big resource consumer. The R&D and other business activities(U35) had the highest PageRank in 2017. However, it had a low evaluation value. This shows that although China has begun to focus on research and development, it will take some time for the technology industry to become a pillar industry in China.
4. The industry gap is narrowing, but it is still large. The biggest difference of industrial comprehensive evaluation value is 0.77, 0.73, 0.80, 0.72 respectively in 2002, 2007, 2012, and 2017. Although the trend of the industrial gap is decreasing, the value remains around 0.7. The proportion of the service industry and science and technology industry should be increased.

## Conclusion

In this study, a dynamic industry network model was developed to evaluate critical industries based on input-output data from China in 2002, 2007, 2012, and 2017. We analyzed the overall structure of the industry based on network density, network aggregation, and network efficiency. Then, the industry evaluation system was constructed based on degree centrality, betweenness centrality, PageRank, influence coefficient, and induction coefficient. The EW method was used to calculate measurement weight, TOPSIS method was used to evaluate the importance of industries.

To optimize the industrial structure of China, the following management suggestions are put forward. Firstly, the network density and aggregation of industry structures have been declining. However, the network efficiency reveals an increasing trend. We suggest that the country should pay more attention to the formation of industrial clusters and industrial chains, in the process of economic development. The government should formulate more policies to encourage exchanges and cooperation between industries. Secondly, we found that Manufacturing and Industry have high evaluation value at all times. Meanwhile, the values of each measurement of manufacturing and industry were also high. Thus, we should coordinate the allocation of resources among industries, promote the transfer of resources and information among industries, and raise the status of high-tech industries. Meanwhile, we should increase investment in and support for high-tech industries and service-oriented industries.

Although this study used an integrated MCDM method to effectively identify critical industries, there remain to exist some limitations. First, we selected some indicators for measurement, but still, some indicators such as network connectedness and eccentricity were excluded in the study. More indicators could be used in future studies. In addition, this paper studies the dynamic change of industrial structure, but due to the data limitation in which the input-output table is once every five years, it cannot reflect the evolution process of industrial structure. Third, the EW method and TOPSIS method are used in the study, but more methods can be used, such as FUCOM, LBWA, etc. Comparing the results of different methods and selecting the optimal method will be an important direction that deserves our future research and complement the current study.

## Supporting information

S1 Appendix (PDF)

S1 File Sample data. (RAR)
