# Supplementary material for: A social network analysis in dynamic evaluate critical industries based on input-output data of China
Source: PLoS One. 2022 Apr 7;17(4):e0266697. doi: 10.1371/journal.pone.0266697 (PMC8989312; doi:10.1371/journal.pone.0266697)
Supplement: S1 Appendix — (PDF) [file pone.0266697.s001.pdf]

Appendix

Table 8. Basic information of industries mentioned in this study.

Id	Industry Name
U2	Coal mining and dressing
U3	Petroleum and natural gas extraction
U4	Metals mining and dressing
U8	Garments and other fiber products
U10	Printing industry, cultural, educational and sports goods
U11	Coke, refined petroleum products and nuclear fuel
U12	Chemicals products
U13	Non-metallic mineral products
U14	Smelting and pressing of metals
U15	Metal products
U16	Ordinary machinery
U17	Special equipment
U18	Transport equipment
U19	Electric equipment and machinery
U20	Electronic and telecommunications equipment
U21	Instruments and meters
U22	Other manufacturing and scrap waste
U23	Metalwork, machinery and equipment repair services
U24	Production and supply of electric power, steam and hot water
U25	Production and supply of gas
U28	Wholesale and retail
U35	R&D and other business activities
U39	Education
